# Anticancer Platinum Drugs Update

**DOI:** 10.3390/biom11111637

**Published:** 2021-11-04

**Authors:** Giuliano Ciarimboli

**Affiliations:** Medizinische Klinik D, Experimentelle Nephrologie, Universitätsklinikum Münster, 48149 Münster, Germany; gciari@uni-muenster.de

The discovery of the anticancer properties of platinum derivatives by Rosenberg [1,2,3] represents a milestone in the development of chemotherapeutic protocols for tumor treatment. In 1978, cisplatin was the first platinum drug approved by the Food and Drug Administration for the treatment of testicular cancer and advanced ovarian and bladder cancer. Cisplatin is still an important component of combination therapy of solid tumors, such as bladder, cervical, ovarian, lung, gastric, breast, and head and neck cancers. Importantly, the chemotherapeutic treatment of testicular cancer with cisplatin is considered to be almost curative [4]. The platinum derivatives cisplatin, carboplatin, and oxaliplatin are clinically approved worldwide [5], and nearly half of all chemotherapeutic protocols for cancer treatment contain platinum derivatives [5]. Unfortunately, the success of anticancer therapy with platinum containing drugs is limited by the development of resistance and of severe side effects, such as nephrotoxicity (cisplatin), ototoxicity (cisplatin), myelosuppression (carboplatin), and peripheral neurotoxicity (oxaliplatin) [6]. These toxicities are dose-limiting and strongly decrease quality of life in cancer survivors. Therefore, to avoid the development of resistance and toxic, unwanted effects, other platinum derivatives and application strategies have been developed and are still in development. Cisplatin and other platinum derivatives damage cancer cells by binding to DNA and forming adducts, which produce distortions in the double helix, affecting DNA transcription and replication and inducing cell apoptosis. Cancer cells are especially sensitive to this toxic action of platinum-containing compounds, since they cannot properly repair the DNA, and die [7]. However, only a small fraction of platinum-containing drugs that enter the cell bind to DNA. The rest exerts an anticancer action by binding to other cellular components and modulating the activity of the immune system [8].

The aim of this Special Issue was to offer an update on the role of platinum drugs in modern cancer therapy, based on expert contributions in this field.

Five contributions were published in this Special Issue, including one review and four original research articles.

The review by Cédric Rébé et al. [9] focuses on the antitumor immune response and the reaction induced by platinum-based anticancer drugs. These drugs can enhance or even induce immunogenic cancer cell death (ICD). The induction of ICD has the potential to eradicate cancer cells. Beside describing the methods used to detect ICD in in vitro and in vivo experiments, the authors of this review have introduced oxaliplatin as the platinum-containing drug with the greatest ability to induce ICD. The role of platinum derivatives as modulators of antitumor immune response and facilitators of immune checkpoint inhibitors’ efficacy is also discussed.

The work by Frenzel et al. [10] focuses on the human organic cation transporters 2 (hOCT2), which is the transporter that likely mediates the uptake of cisplatin and oxaliplatin in the kidneys [11], cochlea [12], and dorsal root ganglia [13]. The hOCT2, therefore, plays an important role in mediating the unwanted, toxic effects of cisplatin and oxaliplatin. The authors of this work demonstrated that the Single Nucleotide Polymorphism Ala270Ser (rs316019) can slightly change the function and regulation of hOCT2. Compared with the hOCT2 wildtype, the plasma membrane expression, cisplatin transport, and cisplatin-associated toxicity of hOCT2 Ala270Ser were significantly lower. These results explain the observation that children bearing this hOCT2 mutation are less prone to developing ototoxicity after therapy with cisplatin [14].

As outlined above, the development of resistance to cisplatin, carboplatin, and oxaliplatin is a problem in the chemotherapeutic treatment of cancer. For this reason, other platinum derivatives, such as [Pt(O,O′-acac)(γ-acac)(dimethyl sulfide (DMS))], have been developed. Antonaci et al. [15] compared the cellular effects of treatment of renal Caki-1 cells with [Pt(O,O′-acac)(γ-acac) (DMS)] and cisplatin. Cisplatin cytotoxicity was low and mediated by apoptosis, while the cytotoxic effect of [Pt(O,O′-acac)(γ-acac)(DMS)] was strong, due to autophagy, which was activated through a mechanism mediated by JNK and PI3K/AKT/mTOR/p70S6K pathways.

Due to the known problems of resistance development and the development of severe side effects, platinum drug delivery strategies using biocompatible transport systems (nanocontainers such as metallic gold, covalent silicon, and silica) to target cancer cells are investigated. For this reason, Nejad and Urbassek [16], using molecular dynamics simulations, studied the adsorption and diffusion of cisplatin molecules in metallic gold, covalent silicon, and silica nanocontainers. They found that the adsorption behavior of cisplatin molecules to the pore walls of nanocontainers is influenced by both van der Waals forces and electrostatic interactions. The van der Waals forces reduce the diffusion coefficient of cisplatin molecules, while fluctuations in the electrostatic energy induced by orientation changes in the cisplatin molecule were found to help desorb the molecule from the wall. The authors concluded that both crystalline and amorphous nanoporous silica could be used as cisplatin nanocontainers for drug delivery.

Nguyen et al. [17] investigated the delivery systems for carboplatin. The authors loaded carboplatin on a polyamidoamine (PAMAM) dendrimer, with or without modification by the addition of methoxypolyethylene glycol (mPEG), to decrease toxicity. The chemical structure, size, and morphology of the obtained products were characterized by proton nuclear magnetic resonance, Fourier transform infrared spectroscopy, transmission electron microscopy, and gel permeation chromatography. The cellular toxicity was also measured. The loading of PAMAM-mPEG with carboplatin resulted in high entrapment and a controlled release of carboplatin. Carboplatin release was safe and inhibited the proliferation of cancer cells.

To conclude, this Special Issue describes important findings related to mechanisms that improve the anticancer action of platinum drugs, avoiding unwanted toxicities. All these findings broaden the knowledge of and impact on the pharmacological and clinical applications of different platinum-derived chemotherapeutic drugs.
